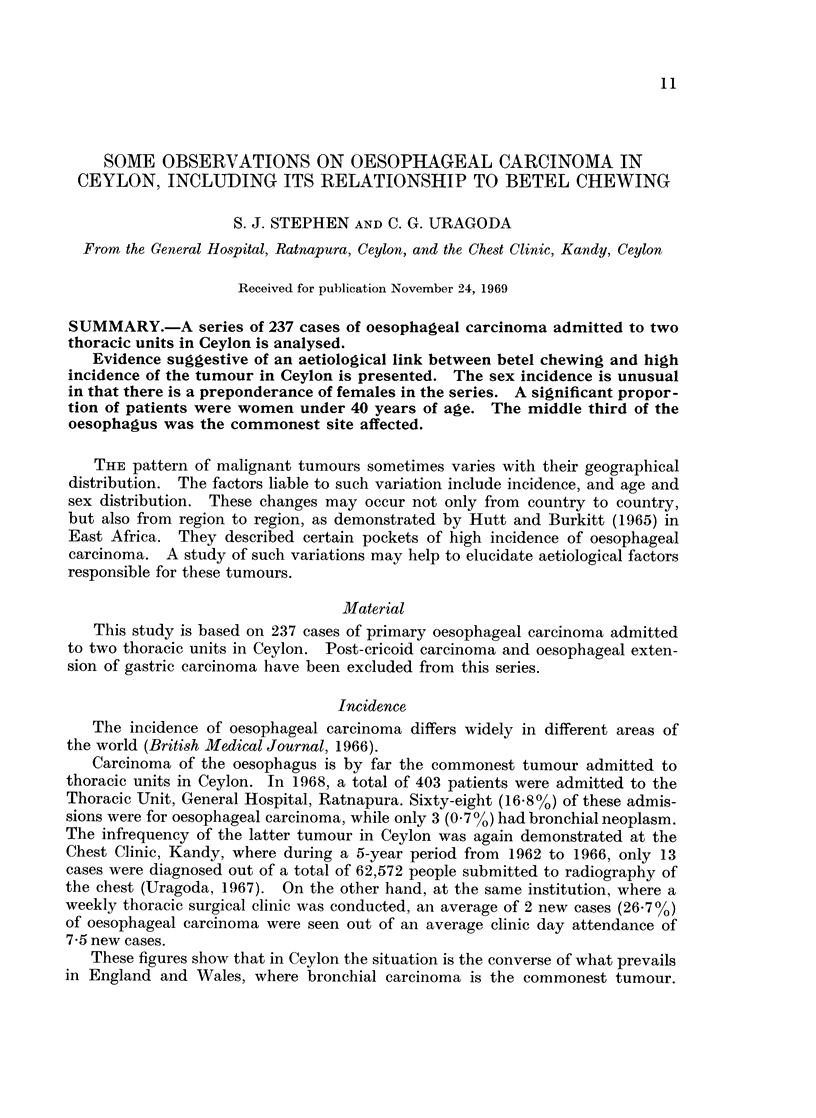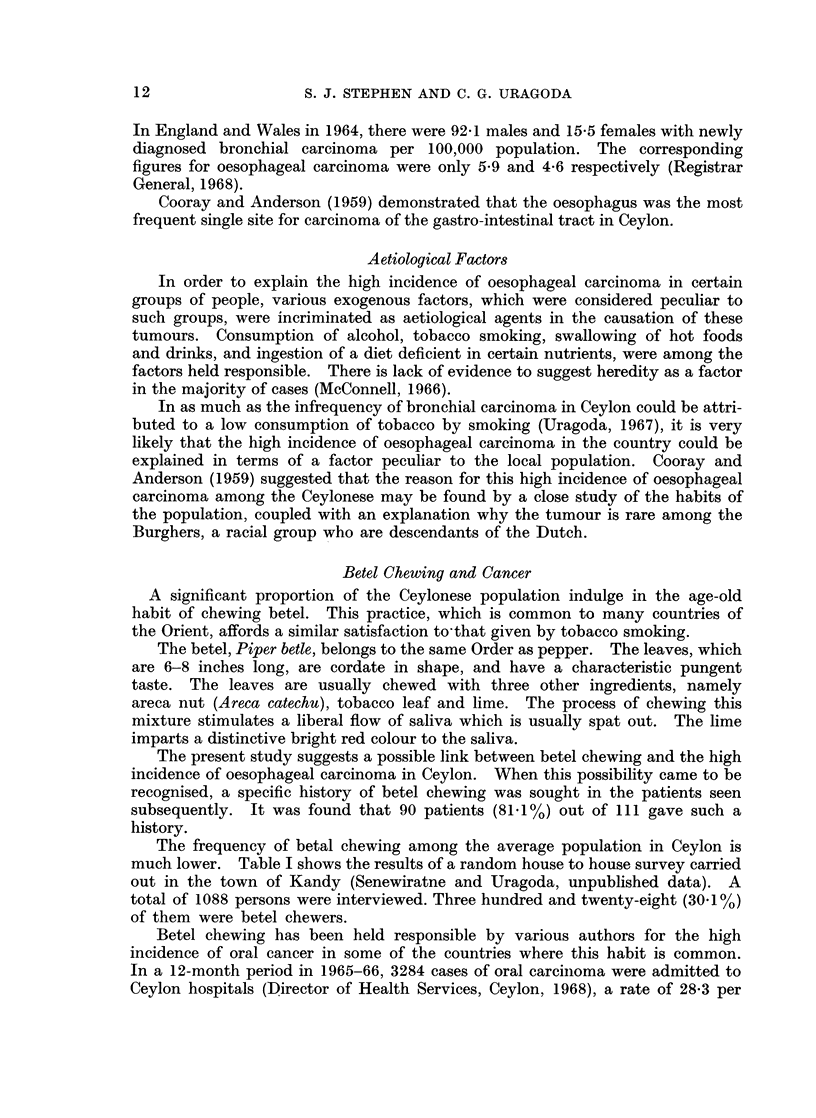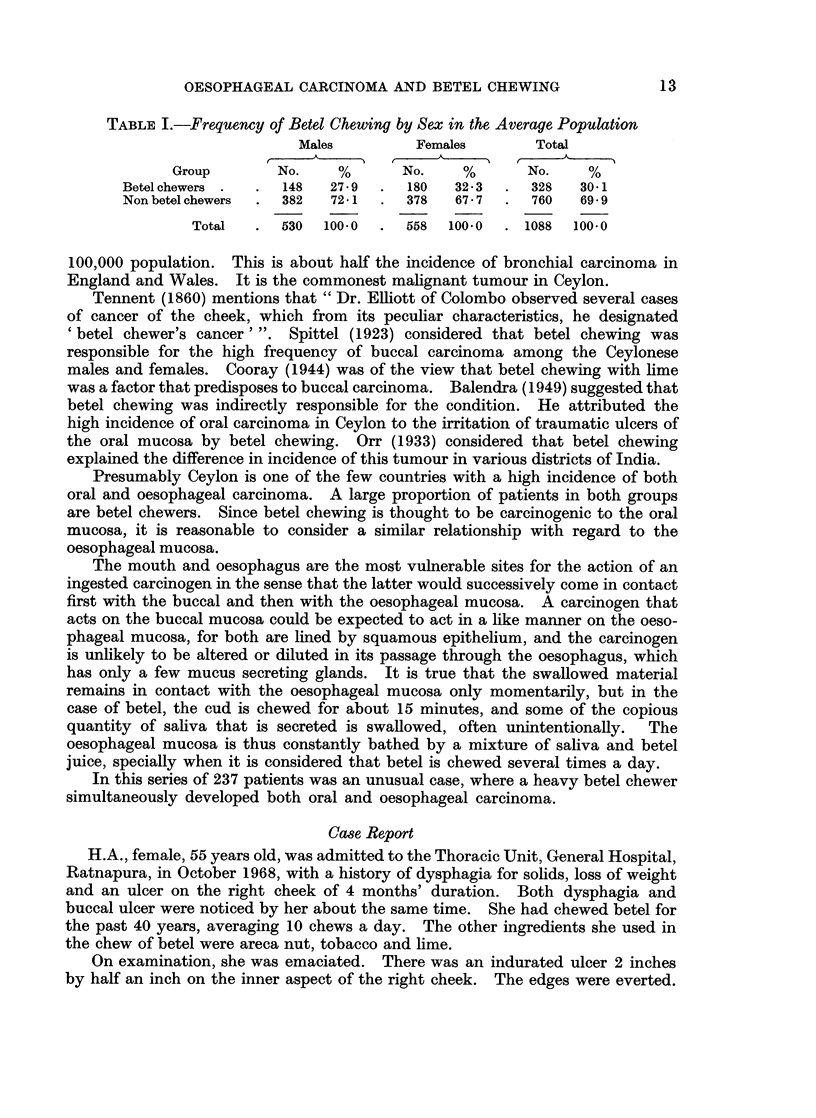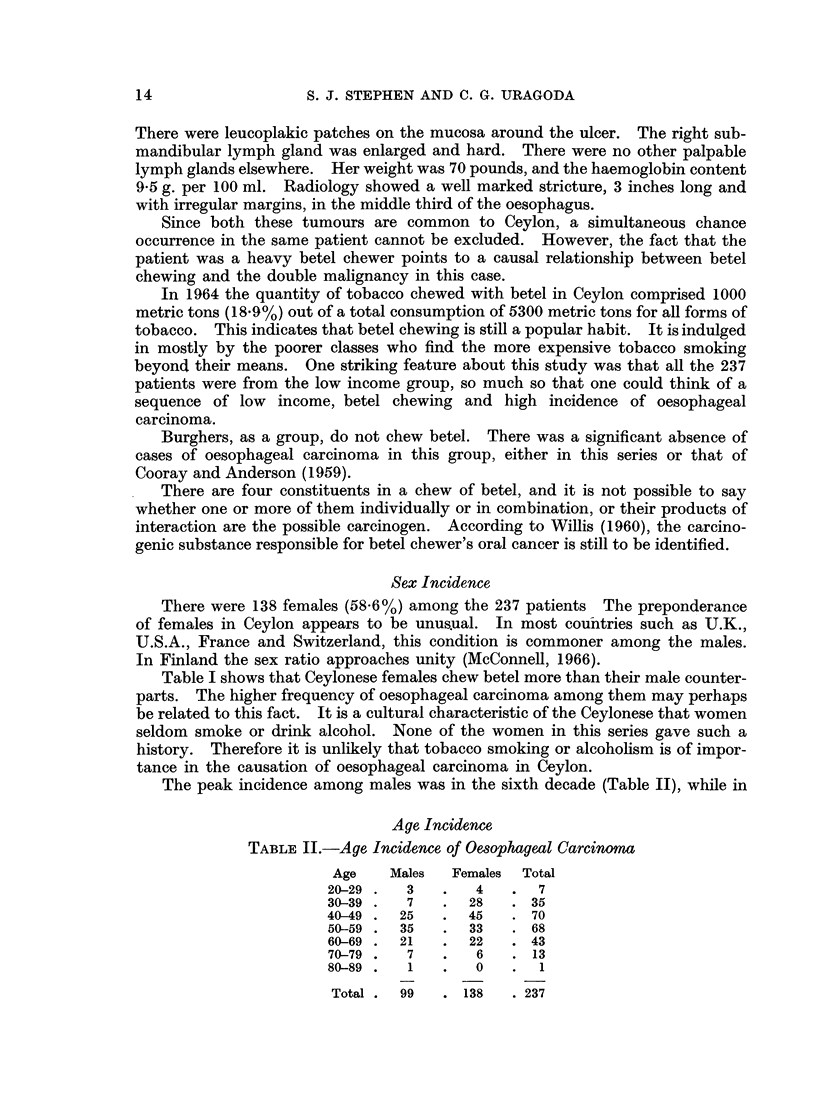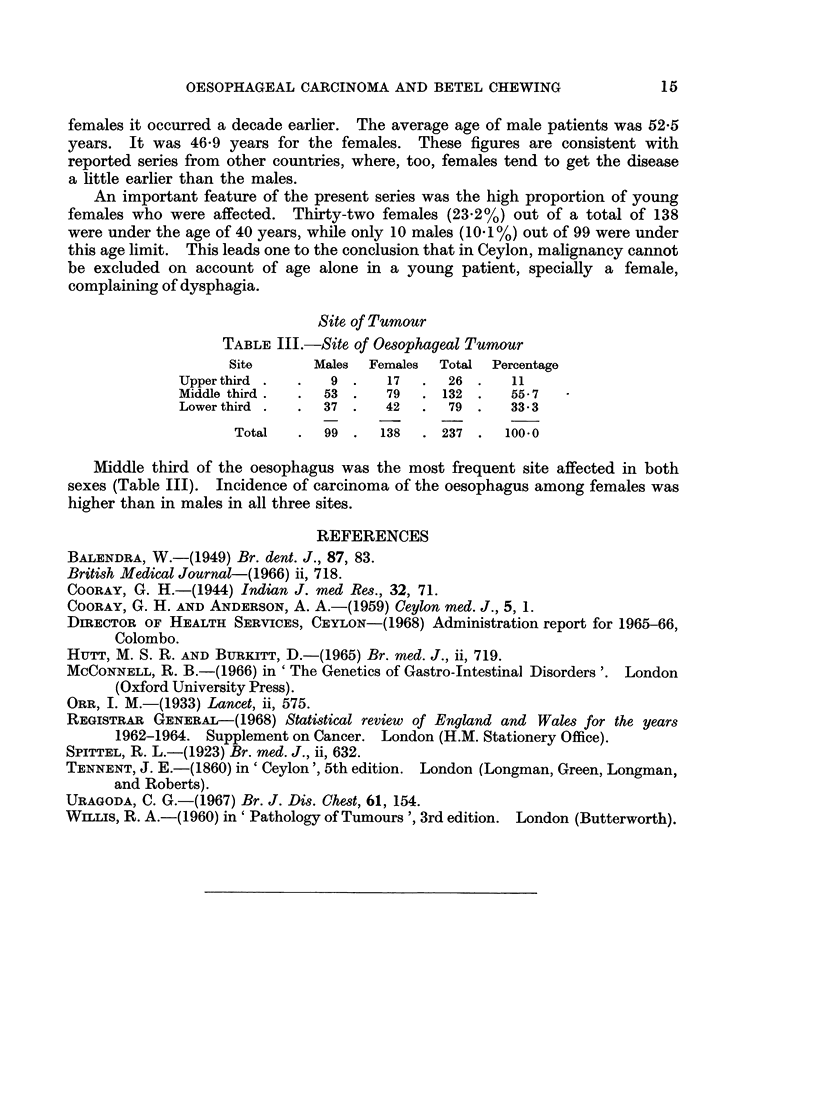# Some Observations on Oesophageal Carcinoma in Ceylon, Including its Relationship to Betel Chewing

**DOI:** 10.1038/bjc.1970.2

**Published:** 1970-03

**Authors:** S. J. Stephen, C. G. Uragoda

## Abstract

A series of 237 cases of oesophageal carcinoma admitted to two thoracic units in Ceylon is analysed.

Evidence suggestive of an aetiological link between betel chewing and high incidence of the tumour in Ceylon is presented. The sex incidence is unusual in that there is a preponderance of females in the series. A significant proportion of patients were women under 40 years of age. The middle third of the oesophagus was the commonest site affected.


### SOME OBSERVATIONS ON OESOPHAGEAL CARCINOMA IN CEYLON, INCLUDING ITS RELATIONSHIP TO BETEL CHEWING

S. J. STEPHEN AND C. G. URAGODA

*From the General Hospital, Ratnapura, Ceylon, and the Chest Clinic, Kandy, Ceylon*

Received for publication November 24, 1969

**SUMMARY.—A series of 237 cases of oesophageal carcinoma admitted to two thoracic units in Ceylon is analysed.**

**Evidence suggestive of an aetiological link between betel chewing and high incidence of the tumour in Ceylon is presented. The sex incidence is unusual in that there is a preponderance of females in the series. A significant proportion of patients were women under 40 years of age. The middle third of the oesophagus was the commonest site affected.**

THE pattern of malignant tumours sometimes varies with their geographical distribution. The factors liable to such variation include incidence, and age and sex distribution. These changes may occur not only from country to country, but also from region to region, as demonstrated by Hutt and Burkitt (1965) in East Africa. They described certain pockets of high incidence of oesophageal carcinoma. A study of such variations may help to elucidate aetiological factors responsible for these tumours.

### *Material*

This study is based on 237 cases of primary oesophageal carcinoma admitted to two thoracic units in Ceylon. Post-cricoid carcinoma and oesophageal extension of gastric carcinoma have been excluded from this series.

### *Incidence*

The incidence of oesophageal carcinoma differs widely in different areas of the world (*British Medical Journal*, 1966).

Carcinoma of the oesophagus is by far the commonest tumour admitted to thoracic units in Ceylon. In 1968, a total of 403 patients were admitted to the Thoracic Unit, General Hospital, Ratnapura. Sixty-eight (16·8%) of these admissions were for oesophageal carcinoma, while only 3 (0·7%) had bronchial neoplasm. The infrequency of the latter tumour in Ceylon was again demonstrated at the Chest Clinic, Kandy, where during a 5-year period from 1962 to 1966, only 13 cases were diagnosed out of a total of 62,572 people submitted to radiography of the chest (Uragoda, 1967). On the other hand, at the same institution, where a weekly thoracic surgical clinic was conducted, an average of 2 new cases (26·7%) of oesophageal carcinoma were seen out of an average clinic day attendance of 7·5 new cases.

These figures show that in Ceylon the situation is the converse of what prevails in England and Wales, where bronchial carcinoma is the commonest tumour.

In England and Wales in 1964, there were 92·1 males and 15·5 females with newly diagnosed bronchial carcinoma per 100,000 population. The corresponding figures for oesophageal carcinoma were only 5·9 and 4·6 respectively (Registrar General, 1968).

Cooray and Anderson (1959) demonstrated that the oesophagus was the most frequent single site for carcinoma of the gastro-intestinal tract in Ceylon.

### *Aetiological Factors*

In order to explain the high incidence of oesophageal carcinoma in certain groups of people, various exogenous factors, which were considered peculiar to such groups, were incriminated as aetiological agents in the causation of these tumours. Consumption of alcohol, tobacco smoking, swallowing of hot foods and drinks, and ingestion of a diet deficient in certain nutrients, were among the factors held responsible. There is lack of evidence to suggest heredity as a factor in the majority of cases (McConnell, 1966).

In as much as the infrequency of bronchial carcinoma in Ceylon could be attributed to a low consumption of tobacco by smoking (Uragoda, 1967), it is very likely that the high incidence of oesophageal carcinoma in the country could be explained in terms of a factor peculiar to the local population. Cooray and Anderson (1959) suggested that the reason for this high incidence of oesophageal carcinoma among the Ceylonese may be found by a close study of the habits of the population, coupled with an explanation why the tumour is rare among the Burghers, a racial group who are descendants of the Dutch.

### *Betel Chewing and Cancer*

A significant proportion of the Ceylonese population indulge in the age-old habit of chewing betel. This practice, which is common to many countries of the Orient, affords a similar satisfaction to that given by tobacco smoking.

The betel, *Piper betle*, belongs to the same Order as pepper. The leaves, which are 6–8 inches long, are cordate in shape, and have a characteristic pungent taste. The leaves are usually chewed with three other ingredients, namely areca nut (*Areca catechu*), tobacco leaf and lime. The process of chewing this mixture stimulates a liberal flow of saliva which is usually spat out. The lime imparts a distinctive bright red colour to the saliva.

The present study suggests a possible link between betel chewing and the high incidence of oesophageal carcinoma in Ceylon. When this possibility came to be recognised, a specific history of betel chewing was sought in the patients seen subsequently. It was found that 90 patients (81·1%) out of 111 gave such a history.

The frequency of betal chewing among the average population in Ceylon is much lower. Table I shows the results of a random house to house survey carried out in the town of Kandy (Senewiratne and Uragoda, unpublished data). A total of 1088 persons were interviewed. Three hundred and twenty-eight (30·1%) of them were betel chewers.

Betel chewing has been held responsible by various authors for the high incidence of oral cancer in some of the countries where this habit is common. In a 12-month period in 1965–66, 3284 cases of oral carcinoma were admitted to Ceylon hospitals (Director of Health Services, Ceylon, 1968), a rate of 28·3 per

TABLE I.—*Frequency of Betel Chewing by Sex in the Average Population*

| Group | Males | | Females | | Total | |
|---|---|---|---|---|---|---|
| | No. | % | No. | % | No. | % |
| Betel chewers | 148 | 27·9 | 180 | 32·3 | 328 | 30·1 |
| Non betel chewers | 382 | 72·1 | 378 | 67·7 | 760 | 69·9 |
| Total | 530 | 100·0 | 558 | 100·0 | 1088 | 100·0 |

100,000 population. This is about half the incidence of bronchial carcinoma in England and Wales. It is the commonest malignant tumour in Ceylon.

Tennent (1860) mentions that " Dr. Elliott of Colombo observed several cases of cancer of the cheek, which from its peculiar characteristics, he designated ' betel chewer's cancer ' ". Spittel (1923) considered that betel chewing was responsible for the high frequency of buccal carcinoma among the Ceylonese males and females. Cooray (1944) was of the view that betel chewing with lime was a factor that predisposes to buccal carcinoma. Balendra (1949) suggested that betel chewing was indirectly responsible for the condition. He attributed the high incidence of oral carcinoma in Ceylon to the irritation of traumatic ulcers of the oral mucosa by betel chewing. Orr (1933) considered that betel chewing explained the difference in incidence of this tumour in various districts of India.

Presumably Ceylon is one of the few countries with a high incidence of both oral and oesophageal carcinoma. A large proportion of patients in both groups are betel chewers. Since betel chewing is thought to be carcinogenic to the oral mucosa, it is reasonable to consider a similar relationship with regard to the oesophageal mucosa.

The mouth and oesophagus are the most vulnerable sites for the action of an ingested carcinogen in the sense that the latter would successively come in contact first with the buccal and then with the oesophageal mucosa. A carcinogen that acts on the buccal mucosa could be expected to act in a like manner on the oesophageal mucosa, for both are lined by squamous epithelium, and the carcinogen is unlikely to be altered or diluted in its passage through the oesophagus, which has only a few mucus secreting glands. It is true that the swallowed material remains in contact with the oesophageal mucosa only momentarily, but in the case of betel, the cud is chewed for about 15 minutes, and some of the copious quantity of saliva that is secreted is swallowed, often unintentionally. The oesophageal mucosa is thus constantly bathed by a mixture of saliva and betel juice, specially when it is considered that betel is chewed several times a day.

In this series of 237 patients was an unusual case, where a heavy betel chewer simultaneously developed both oral and oesophageal carcinoma.

### *Case Report*

H.A., female, 55 years old, was admitted to the Thoracic Unit, General Hospital, Ratnapura, in October 1968, with a history of dysphagia for solids, loss of weight and an ulcer on the right cheek of 4 months' duration. Both dysphagia and buccal ulcer were noticed by her about the same time. She had chewed betel for the past 40 years, averaging 10 chews a day. The other ingredients she used in the chew of betel were areca nut, tobacco and lime.

On examination, she was emaciated. There was an indurated ulcer 2 inches by half an inch on the inner aspect of the right cheek. The edges were everted.

There were leucoplakic patches on the mucosa around the ulcer. The right submandibular lymph gland was enlarged and hard. There were no other palpable lymph glands elsewhere. Her weight was 70 pounds, and the haemoglobin content 9·5 g. per 100 ml. Radiology showed a well marked stricture, 3 inches long and with irregular margins, in the middle third of the oesophagus.

Since both these tumours are common to Ceylon, a simultaneous chance occurrence in the same patient cannot be excluded. However, the fact that the patient was a heavy betel chewer points to a causal relationship between betel chewing and the double malignancy in this case.

In 1964 the quantity of tobacco chewed with betel in Ceylon comprised 1000 metric tons (18·9%) out of a total consumption of 5300 metric tons for all forms of tobacco. This indicates that betel chewing is still a popular habit. It is indulged in mostly by the poorer classes who find the more expensive tobacco smoking beyond their means. One striking feature about this study was that all the 237 patients were from the low income group, so much so that one could think of a sequence of low income, betel chewing and high incidence of oesophageal carcinoma.

Burghers, as a group, do not chew betel. There was a significant absence of cases of oesophageal carcinoma in this group, either in this series or that of Cooray and Anderson (1959).

There are four constituents in a chew of betel, and it is not possible to say whether one or more of them individually or in combination, or their products of interaction are the possible carcinogen. According to Willis (1960), the carcinogenic substance responsible for betel chewer's oral cancer is still to be identified.

*Sex Incidence*

There were 138 females (58·6%) among the 237 patients The preponderance of females in Ceylon appears to be unusual. In most countries such as U.K., U.S.A., France and Switzerland, this condition is commoner among the males. In Finland the sex ratio approaches unity (McConnell, 1966).

Table I shows that Ceylonese females chew betel more than their male counterparts. The higher frequency of oesophageal carcinoma among them may perhaps be related to this fact. It is a cultural characteristic of the Ceylonese that women seldom smoke or drink alcohol. None of the women in this series gave such a history. Therefore it is unlikely that tobacco smoking or alcoholism is of importance in the causation of oesophageal carcinoma in Ceylon.

The peak incidence among males was in the sixth decade (Table II), while in

*Age Incidence*

TABLE II.—*Age Incidence of Oesophageal Carcinoma*

| Age | Males | Females | Total |
|---|---|---|---|
| 20–29 | 3 | 4 | 7 |
| 30–39 | 7 | 28 | 35 |
| 40–49 | 25 | 45 | 70 |
| 50–59 | 35 | 33 | 68 |
| 60–69 | 21 | 22 | 43 |
| 70–79 | 7 | 6 | 13 |
| 80–89 | 1 | 0 | 1 |
| Total | 99 | 138 | 237 |

females it occurred a decade earlier. The average age of male patients was 52·5 years. It was 46·9 years for the females. These figures are consistent with reported series from other countries, where, too, females tend to get the disease a little earlier than the males.

An important feature of the present series was the high proportion of young females who were affected. Thirty-two females (23·2%) out of a total of 138 were under the age of 40 years, while only 10 males (10·1%) out of 99 were under this age limit. This leads one to the conclusion that in Ceylon, malignancy cannot be excluded on account of age alone in a young patient, specially a female, complaining of dysphagia.

*Site of Tumour*

TABLE III.—*Site of Oesophageal Tumour*

| Site | Males | Females | Total | Percentage |
|---|---|---|---|---|
| Upper third | 9 | 17 | 26 | 11 |
| Middle third | 53 | 79 | 132 | 55·7 |
| Lower third | 37 | 42 | 79 | 33·3 |
| Total | 99 | 138 | 237 | 100·0 |

Middle third of the oesophagus was the most frequent site affected in both sexes (Table III). Incidence of carcinoma of the oesophagus among females was higher than in males in all three sites.

REFERENCES

BALENDRA, W.—(1949) *Br. dent. J.*, **87**, 83.
*British Medical Journal*—(1966) ii, 718.
COORAY, G. H.—(1944) *Indian J. med Res.*, **32**, 71.
COORAY, G. H. AND ANDERSON, A. A.—(1959) *Ceylon med. J.*, **5**, 1.
DIRECTOR OF HEALTH SERVICES, CEYLON—(1968) Administration report for 1965–66, Colombo.
HUTT, M. S. R. AND BURKITT, D.—(1965) *Br. med. J.*, ii, 719.
McCONNELL, R. B.—(1966) in ‘ The Genetics of Gastro-Intestinal Disorders ’. London (Oxford University Press).
ORR, I. M.—(1933) *Lancet*, ii, 575.
REGISTRAR GENERAL—(1968) *Statistical review of England and Wales for the years* 1962–1964. Supplement on Cancer. London (H.M. Stationery Office).
SPITTEL, R. L.—(1923) *Br. med. J.*, ii, 632.
TENNENT, J. E.—(1860) in ‘ Ceylon ’, 5th edition. London (Longman, Green, Longman, and Roberts).
URAGODA, C. G.—(1967) *Br. J. Dis. Chest*, **61**, 154.
WILLIS, R. A.—(1960) in ‘ Pathology of Tumours ’, 3rd edition. London (Butterworth).